# Supplementary material for: Accurate Identification of Spatial Domain by Incorporating Global Spatial Proximity and Local Expression Proximity
Source: Biomolecules. 2024 Jun 9;14(6):674. doi: 10.3390/biom14060674 (PMC11201407; doi:10.3390/biom14060674)
Supplement: Supplementary file 1 [file biomolecules-14-00674-s001.zip › biomolecules-3018716-supplementary.pdf]

Supplementary Figures

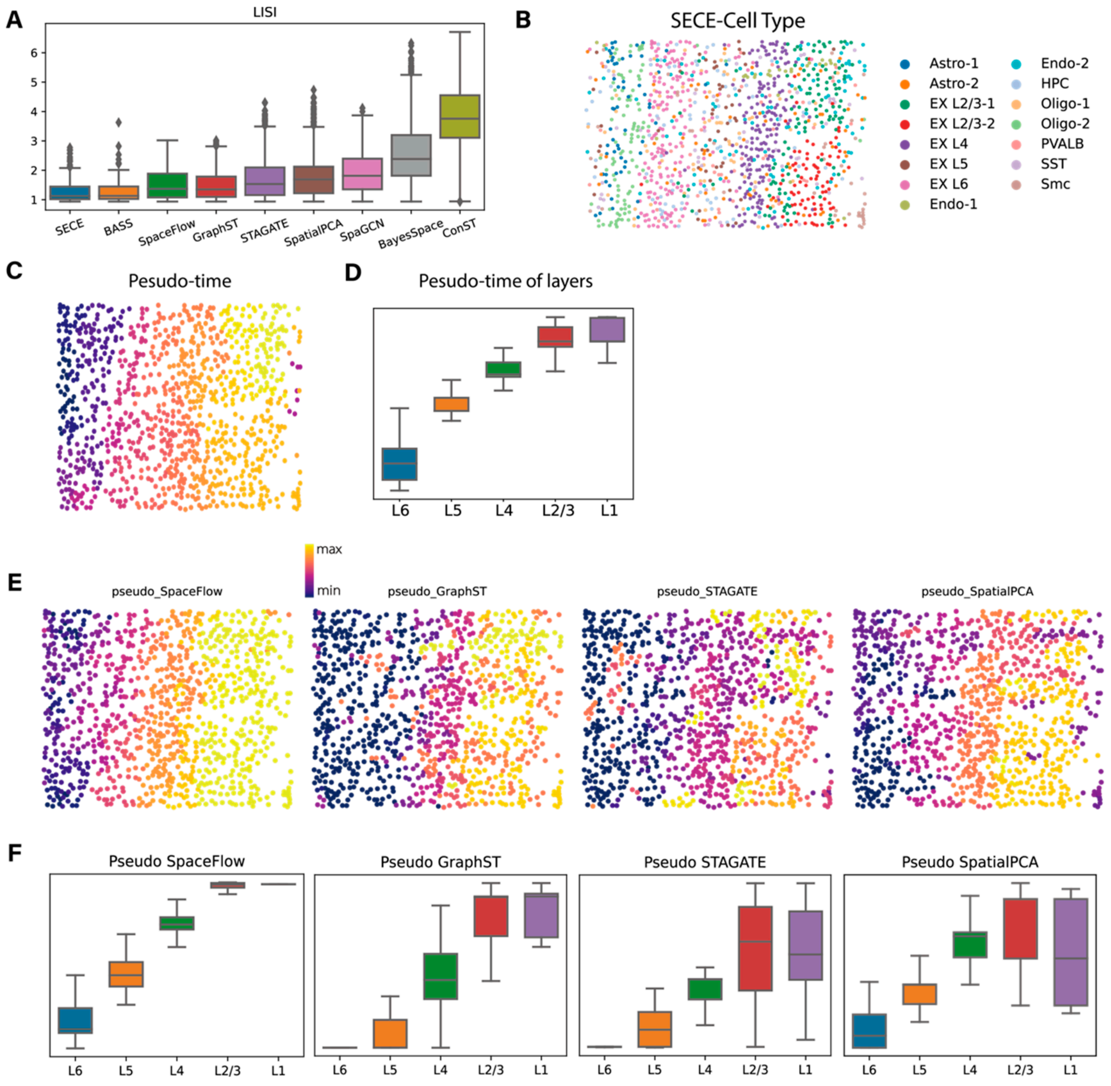

Figure S1. Trajectory inference on mouse visual cortex STARmap data, related to Figure 2. **(A)** Boxplot of LISI measuring spatial aggregation of domains identified by different methods. **(B)** Cell type annotation. Astro, astrocytes; Oligo, oligodendrocytes; EX, excitatory neurons; IN Neuron, inhibitory neurons; Endo, endothelial cells; SMC, smooth muscle cells. **(C)** Pseudo-time of each cell calculated by Monocle3 based on SECE embeddings. **(D)** Pseudo-time of cells in each cortical layer based on SECE. (**E)** Pseudo-time of each cell calculated by Monocle3 based on SpaceFlow, GraphST, STAGATE, and SpatialPCA embeddings. (**F)** Pseudo-time of cells in each cortical layer based on SpaceFlow, GraphST, STAGATE and SpatialPCA.

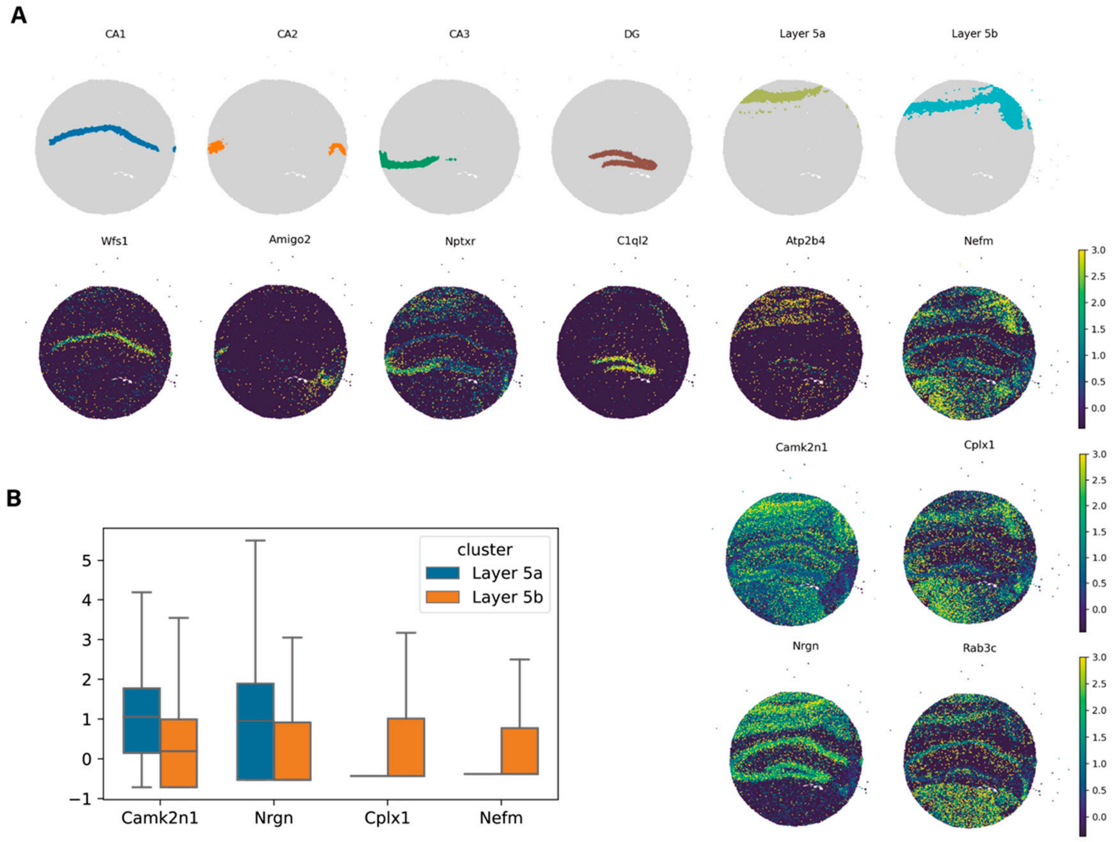

Figure S2. Marker of spatial domains identified by SECE on mouse hippocampus Slide-seqV2 data, related to Figure 3. **(A)** Spatial visualization of CA1, CA2, and CA3 domains identified by SECE (Top) and the corresponding marker genes (Bottom). **(B)** Boxplots showing expression levels of *Camk2n1*, *Nrgn*, *Cplx1*, *Nefm* in Layer 5a and Layer 5b.

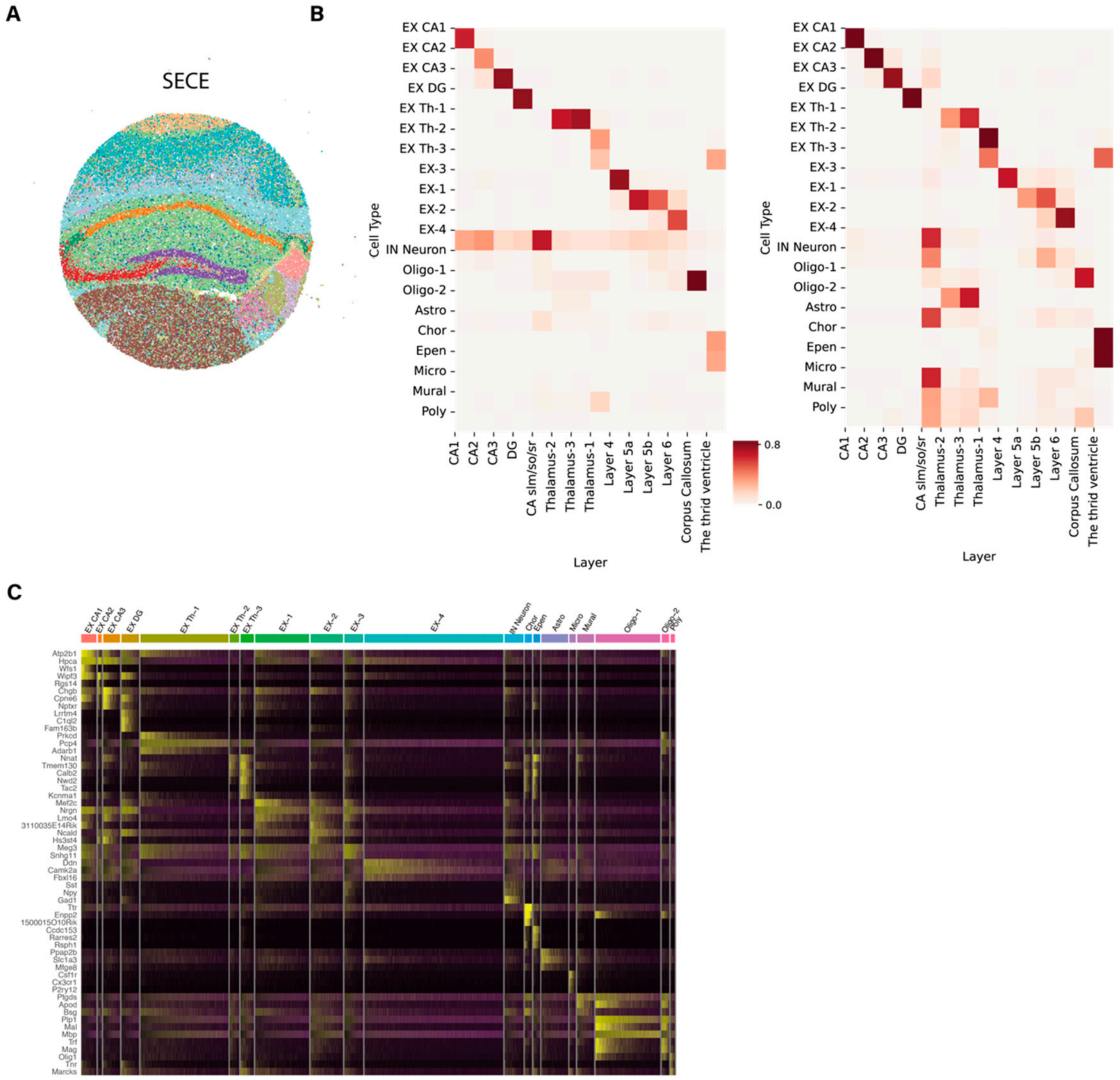

Figure S3. Cell type composition of domains in mouse hippocampus Slide-seqV2 data, related to Figure 3. **(A)** Spatial visualization of cell types. **(B)** Left: Heatmap showing proportion of each cell type across spatial domains identified by SECE. The color represents the proportion of each cell type contained in each spatial region. The sum of the proportions of each spatial domain is 1. Right: Heatmap showing distribution of each cell type across spatial domains. The color represents what proportion of each cell type is distributed in that spatial region. The sum of the proportions of each cell type is 1. **(C)** Gene expression heatmaps for cell type clusters. Each gene was centered and standardized across all the cells. For each cell type cluster, gene expression of the top three differentially expressed (DE) genes are displayed, where DE genes were identified using the Wilcoxon rank-sum test contrasting each cluster of cells against all the remaining cells. Cell type clusters were annotated with specific cell types by comparing the identified DE genes with previously known cell type marker genes.

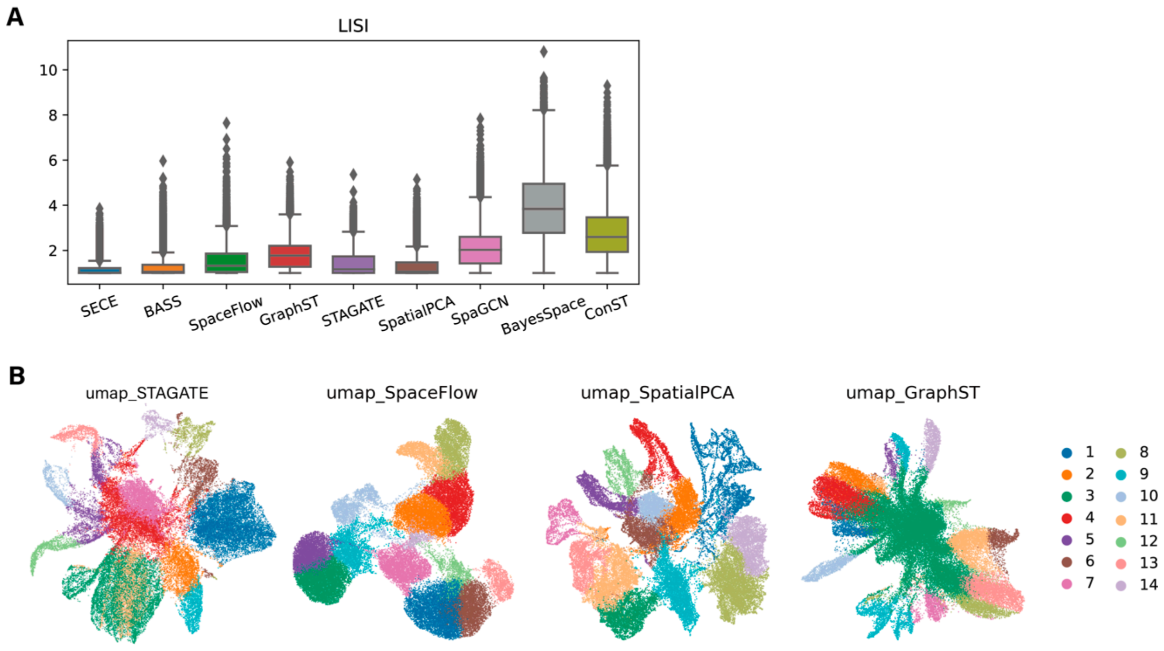

Figure S4. Spatial domains of mouse hippocampus Slide-seqV2 data, related to Figure 3. **(A)** Boxplot of LISI measuring spatial aggregation of domains identified by different methods. **(B)** UMAP visualizations generated by SpaceFlow, GraphST, STAGATE, and SpatialPCA, colored by corresponding domains.

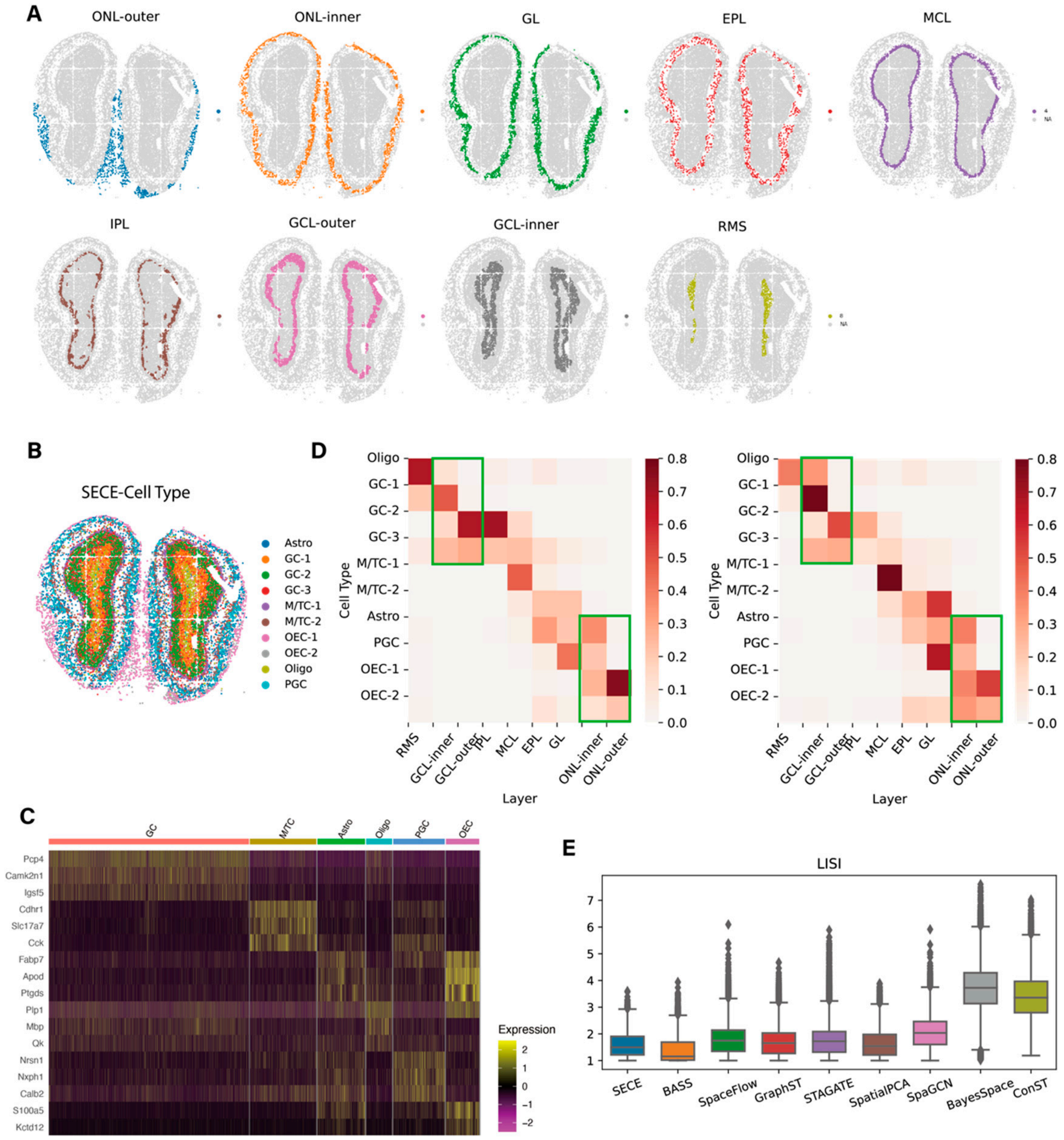

Figure S5. Spatial domain identification of olfactory bulb, related to Figure 4. **(A)** Separate spatial visualization for each identified layer. **(B)** Spatial visualization of cell types. GC, Granule cells; M/TC, Mitral and tufted cells; PGC, Periglomerular cells; OEC, Olfactory ensheathing cells. **(C)** Gene expression heatmaps for cell type clusters. Each gene was centered and standardized across all the cells. For each cell type cluster, gene expression of the top three differentially expressed (DE) genes are displayed, where DE genes were identified using the Wilcoxon rank-sum test contrasting each cluster of cells against all the remaining cells. Cell type clusters were annotated with specific cell types by comparing the identified DE genes with previously known cell type marker genes. **(D)** Left: Heatmap showing proportion of each cell type across spatial domains. The color represents the proportion of each cell type contained in each spatial region. The sum of the proportions of each

spatial domain is 1. Right: Heatmap showing distribution of cell types across spatial domains. The color represents what proportion of each cell type is distributed in that spatial region. The sum of the proportions of each cell type is 1. **(E)** Boxplot of LISI measuring spatial aggregation of domains identified by different methods.

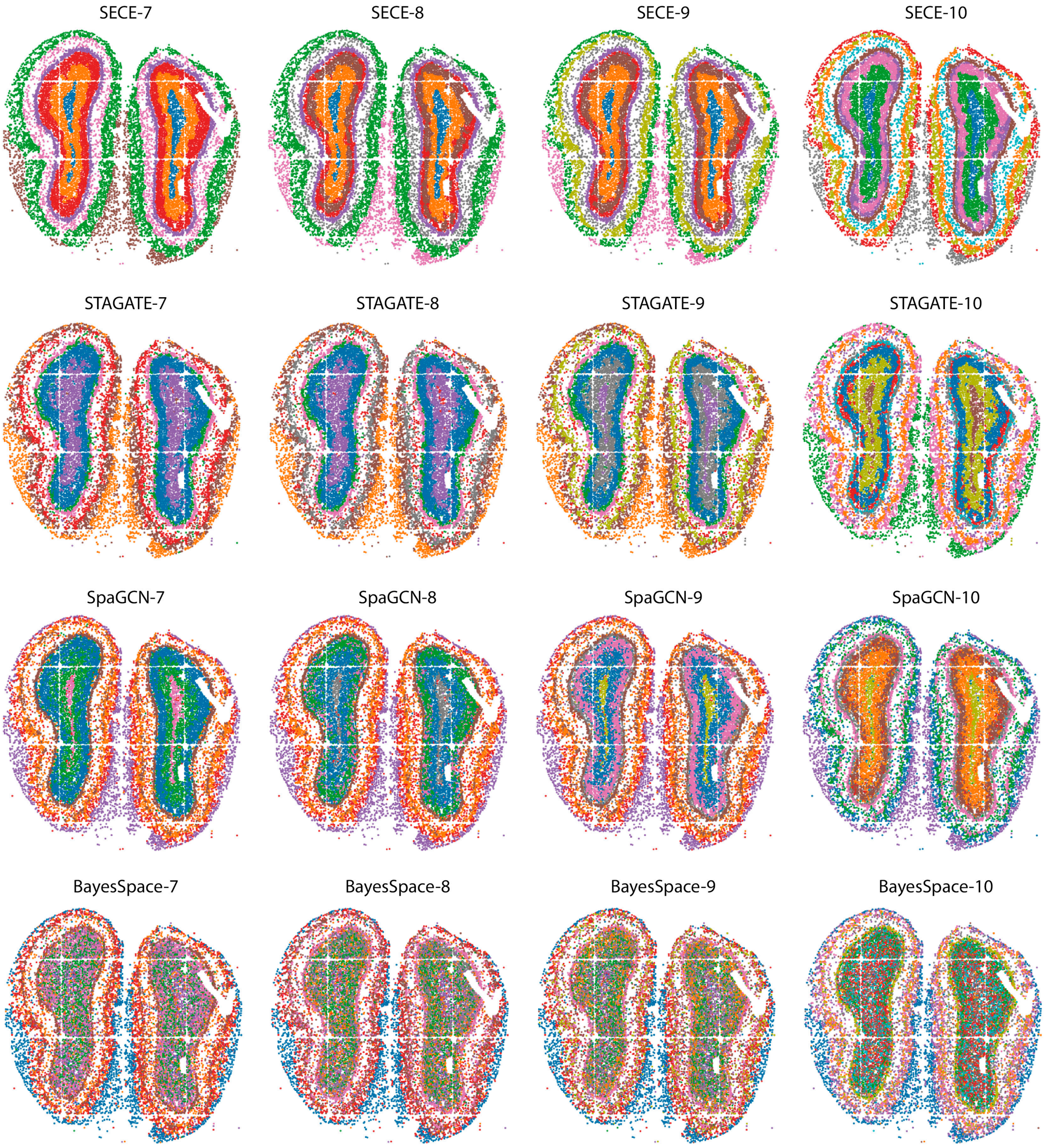

Figure S6. Different number (7, 8, 9, 10) of spatial domains identified by the SECE, STAGATE, SpaGCN and BayesSpace in the Stereo-seq mouse olfactory bulb data.

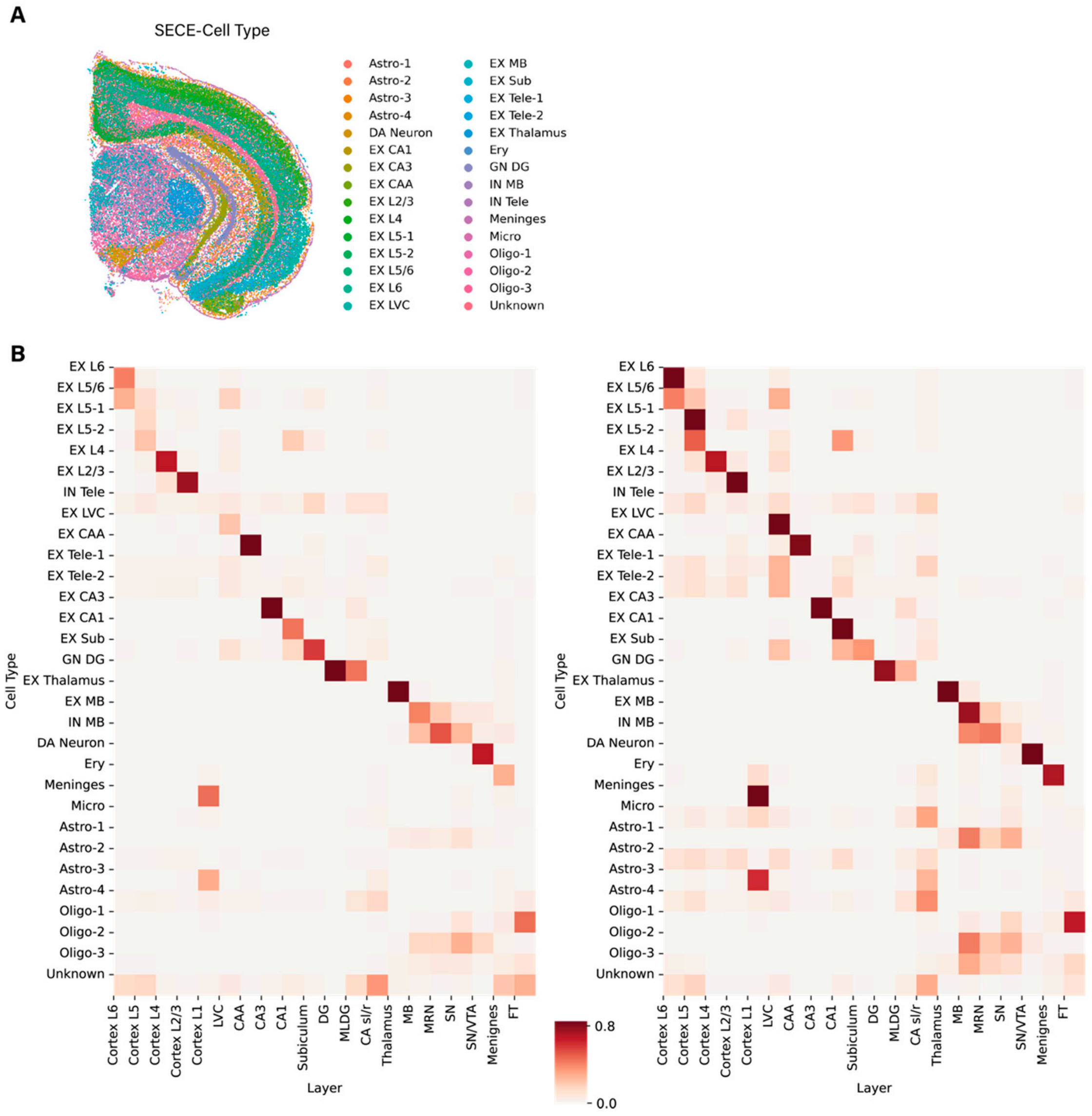

Figure S7. Relationship between cell types and spatial regions identified by SECE of mouse brain Stereo-seq data, related to Figure 5. **(A)** Spatial visualization of cell types. **(B)** Left: Heatmap showing proportion of each cell type across spatial domains identified by SECE. The color represents the proportion of each cell type contained in each spatial region. The sum of the proportions of each spatial domain is 1. Right: Heatmap showing distribution of each cell type across spatial domains identified by SECE. The color represents what proportion of each cell type is distributed in that spatial region. The sum of the proportions of each cell type is 1.

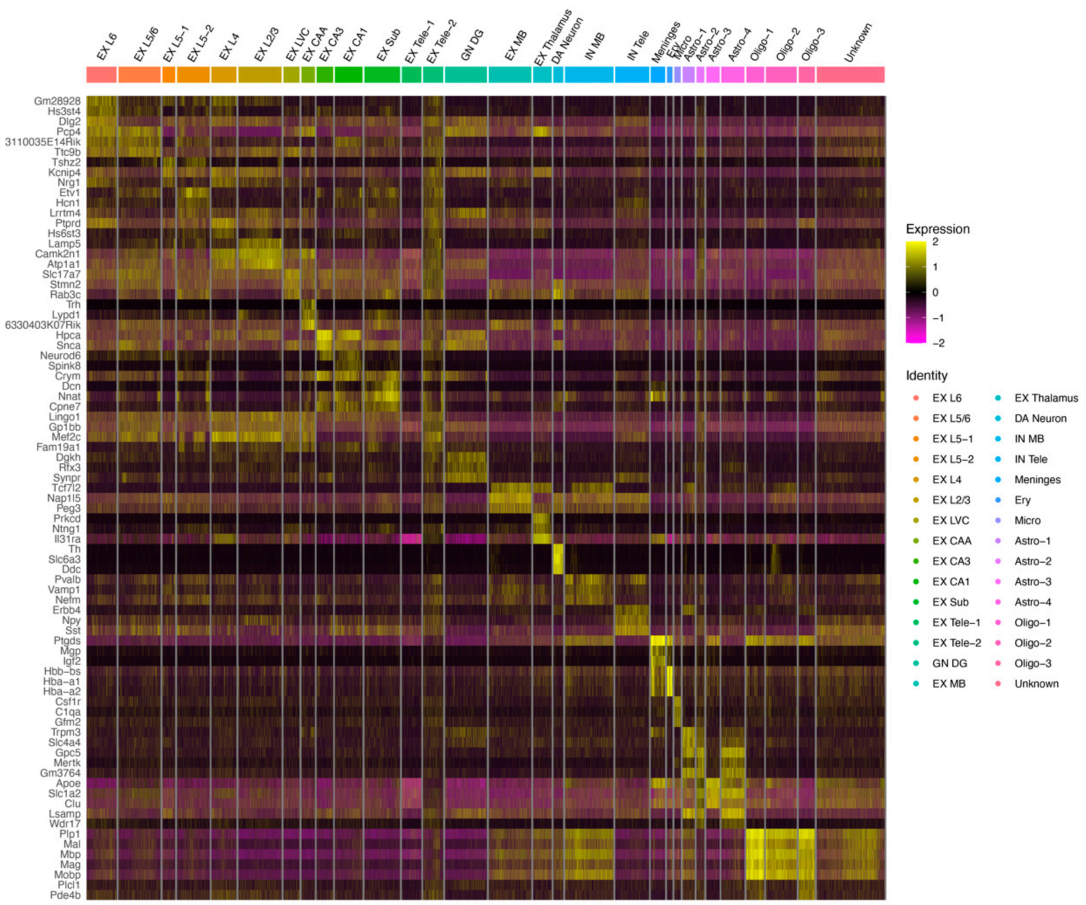

Figure S8. Gene expression heatmaps of cell type clusters in mouse brain Stereo-seq data, related to Figure 5. Each gene was centered and standardized across all the cells. For each cell type cluster, gene expression of the top three differentially expressed (DE) genes are displayed, where DE genes were identified using the Wilcoxon rank-sum test contrasting each cluster of cells against all the remaining cells. Cell type clusters were annotated with specific cell types by comparing the identified DE genes with previously known cell type marker genes.

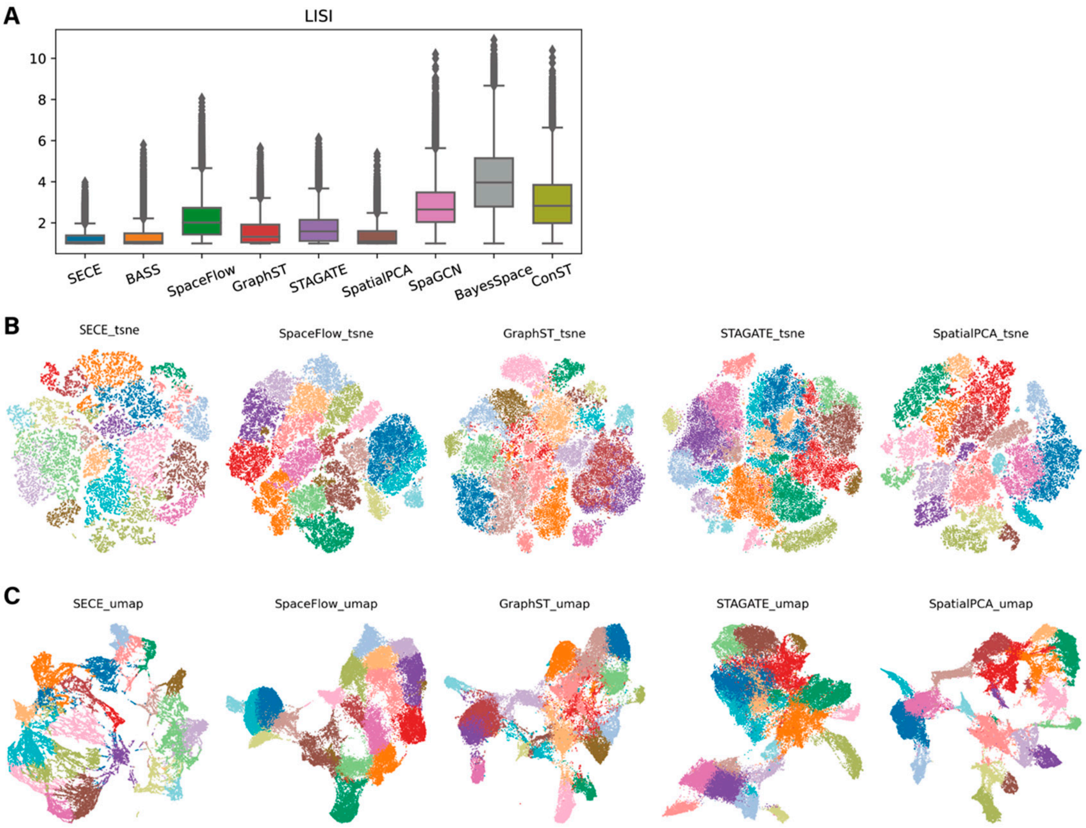

Figure S9. Spatial domain identification of mouse brain Stereo-seq data, related to Fig.5. (**A**) Boxplot of LISI measuring spatial aggregation of domains identified by different methods. (**B**) t-SNE visualizations generated by SECE, SpaceFlow, GraphST, STAGATE, and SpatialPCA embeddings, colored by corresponding spatial domains. **(C)** UMAP visualizations generated by SECE, SpaceFlow, GraphST, STAGATE, and SpatialPCA embeddings, colored by corresponding spatial domains.

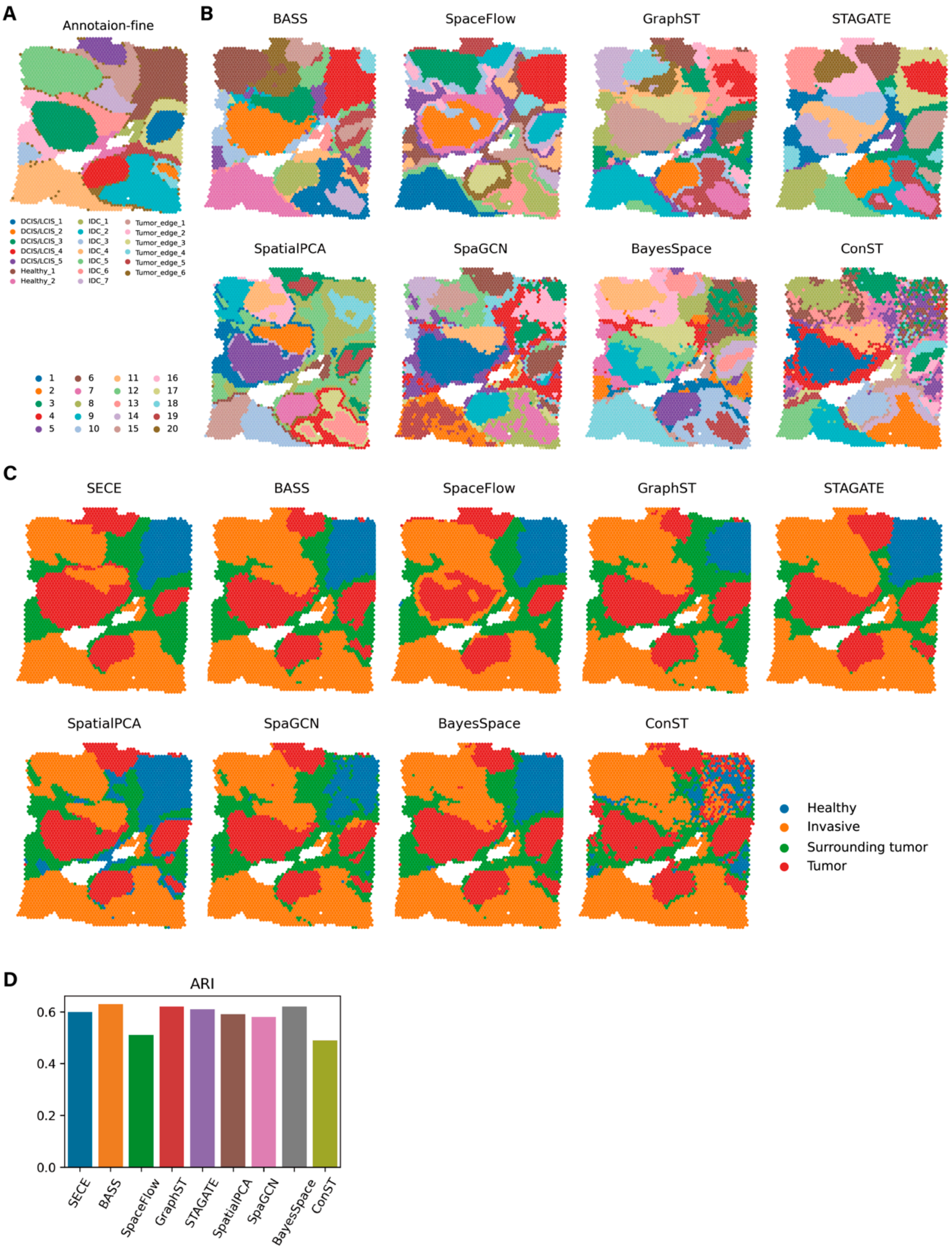

Figure S10. Spatial domain identification of breast cancer data, related to Figure 6. **(A)** Pathology annotation of the tissue section from the original study. **(B)** Spatial regions identified by different methods. **(C)** Annotation of Spatial regions identified by different methods. **(D)** Spatial domain identification performance of different methods measured by ARI.

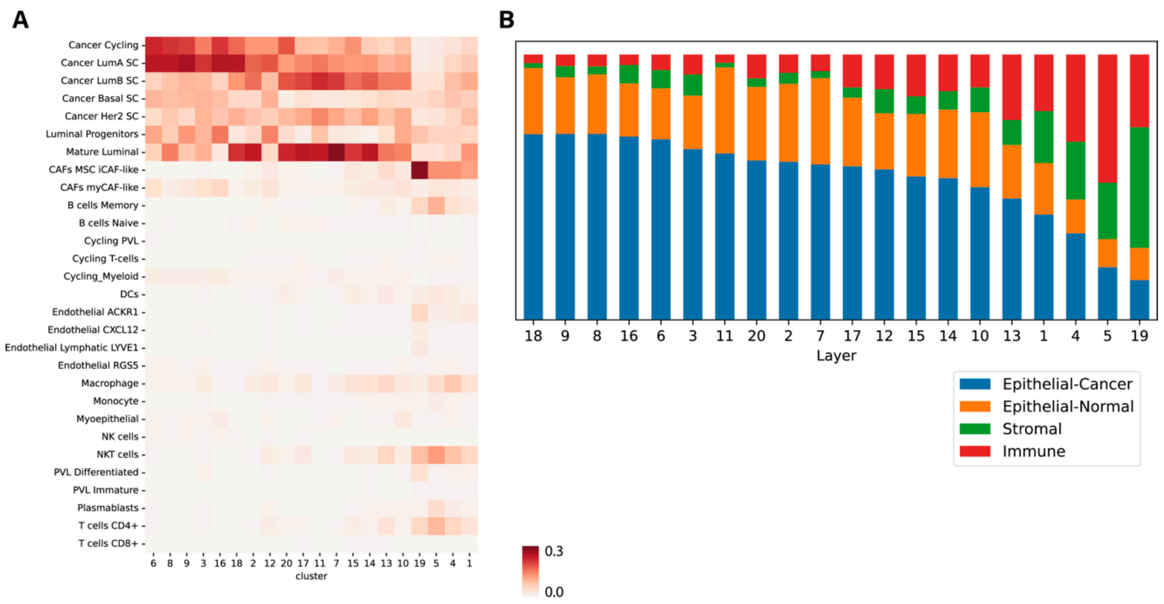

Figure S11. Cell type composition in spatial regions identified by SECE, related to Fig.6. **(A)** Heatmap showing proportion of each cell type across spatial domains identified by SECE, with cell types inferred by cell2location. (**B)** Stacked bar chart showing proportion of summarized cell types across spatial domains. The four cell types are consolidated from the cell2location predictions.

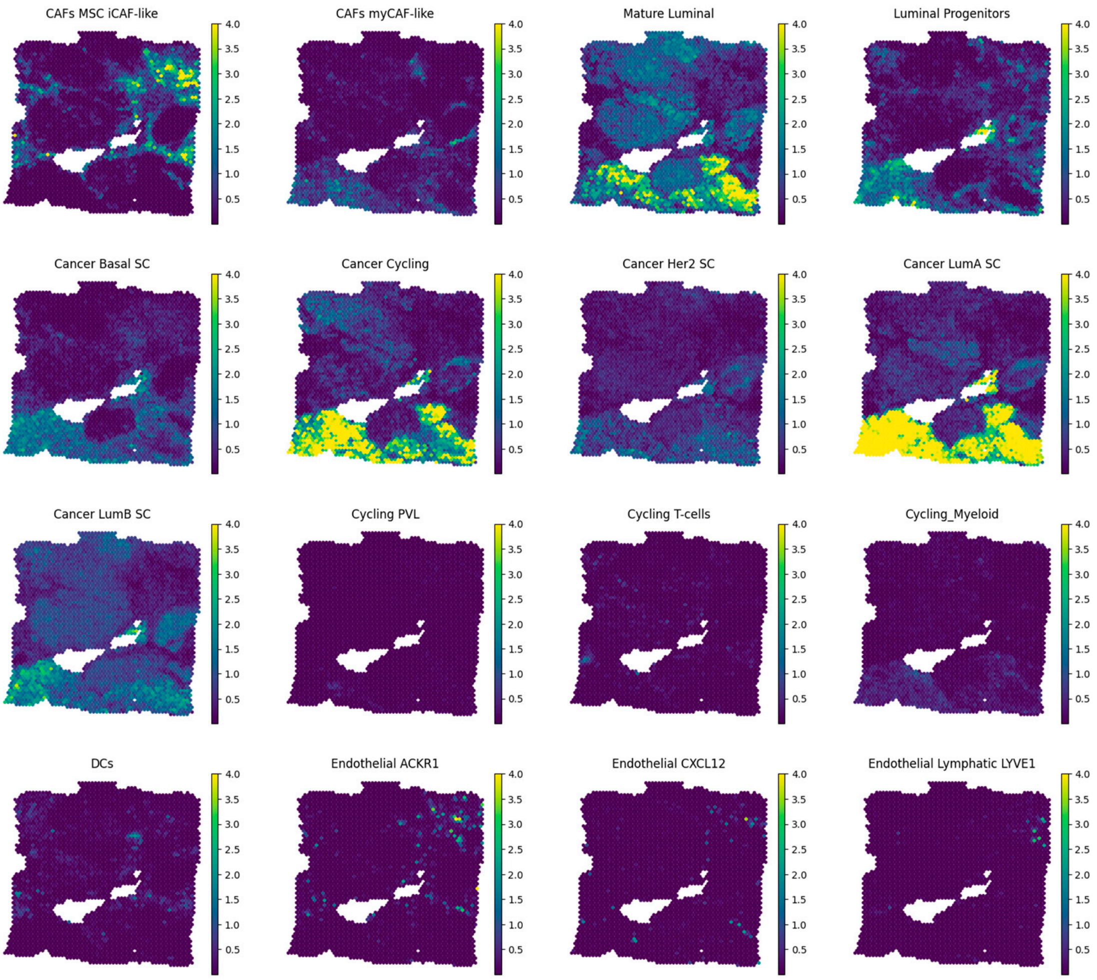

See next page

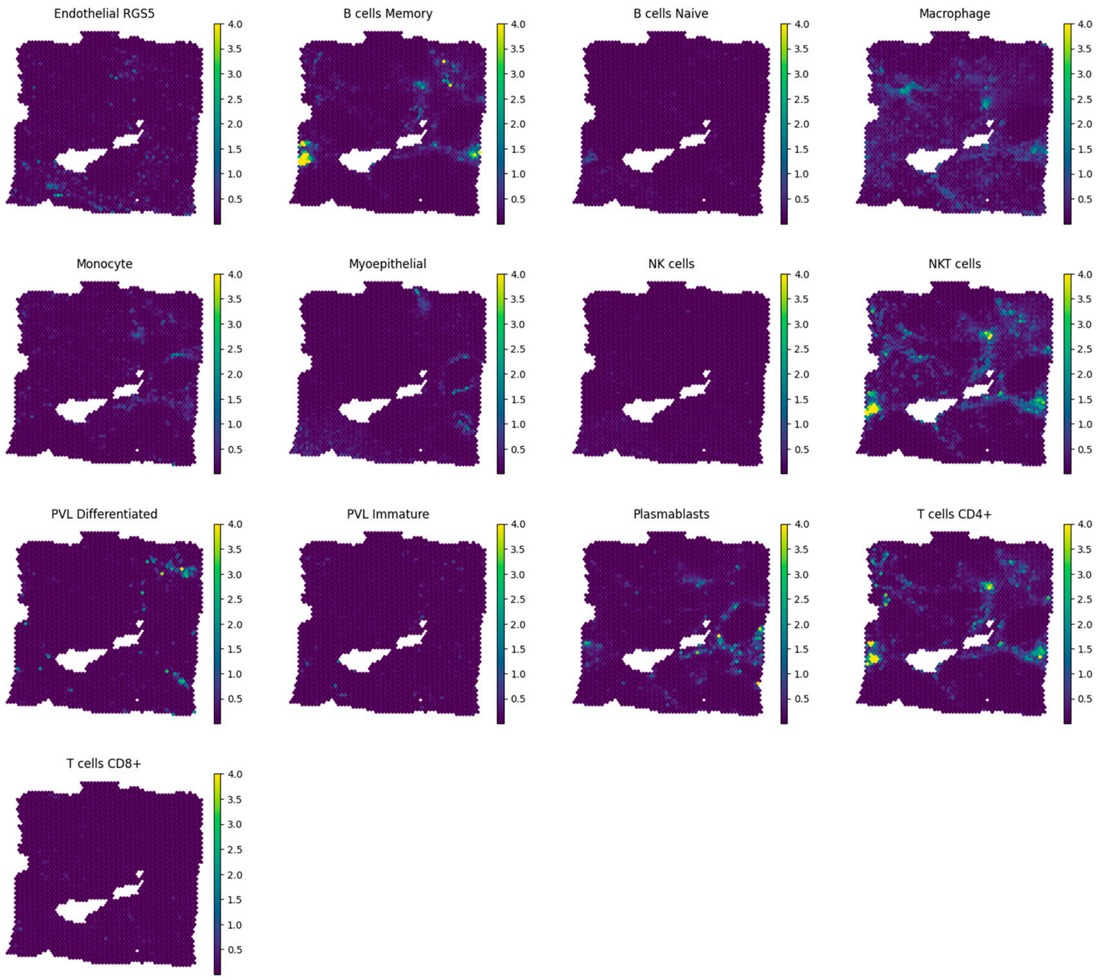

Figure S12. Number of cells per spot for each cell type inferred by cell2location in human breast cancer Visium data.

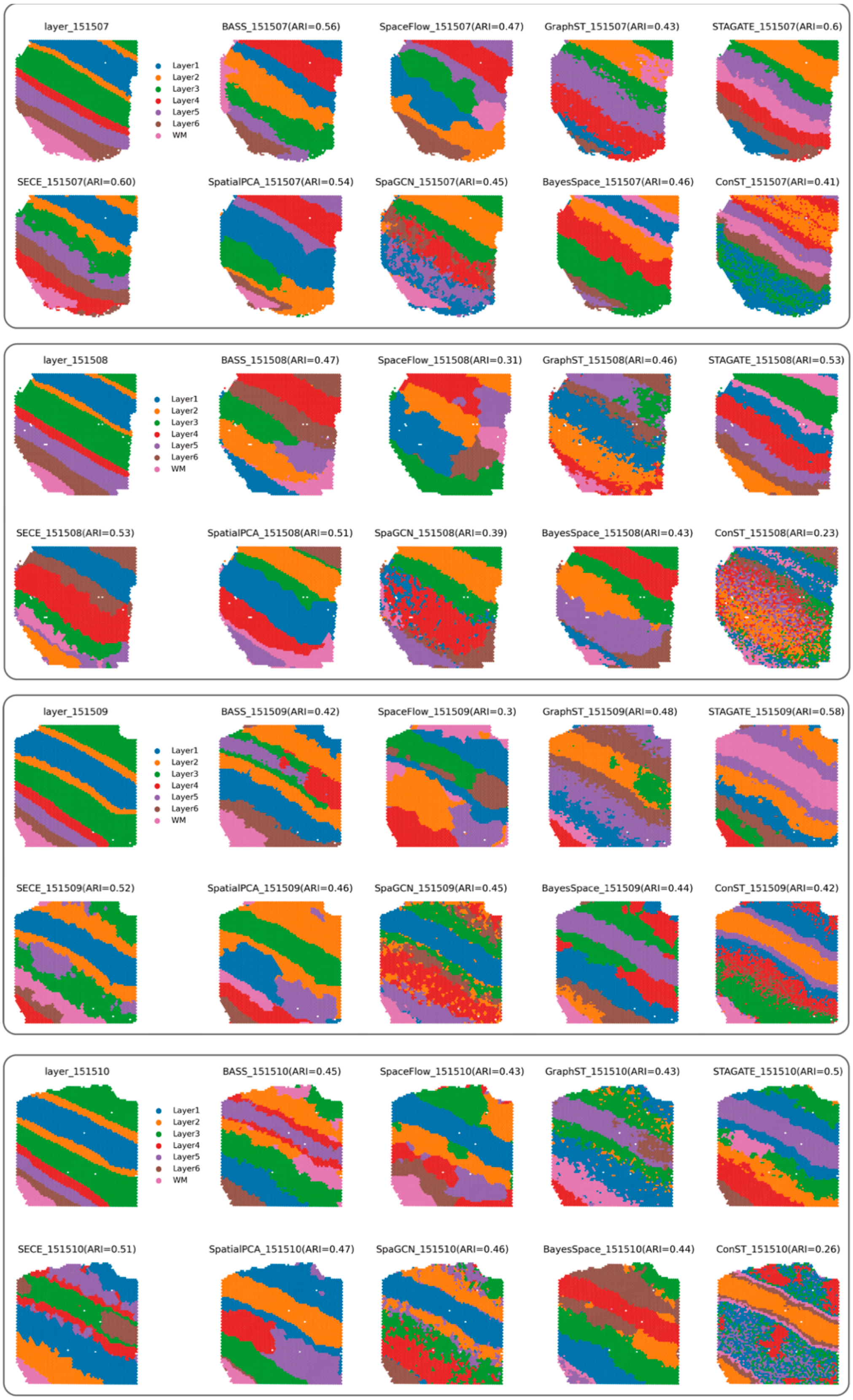

See next page

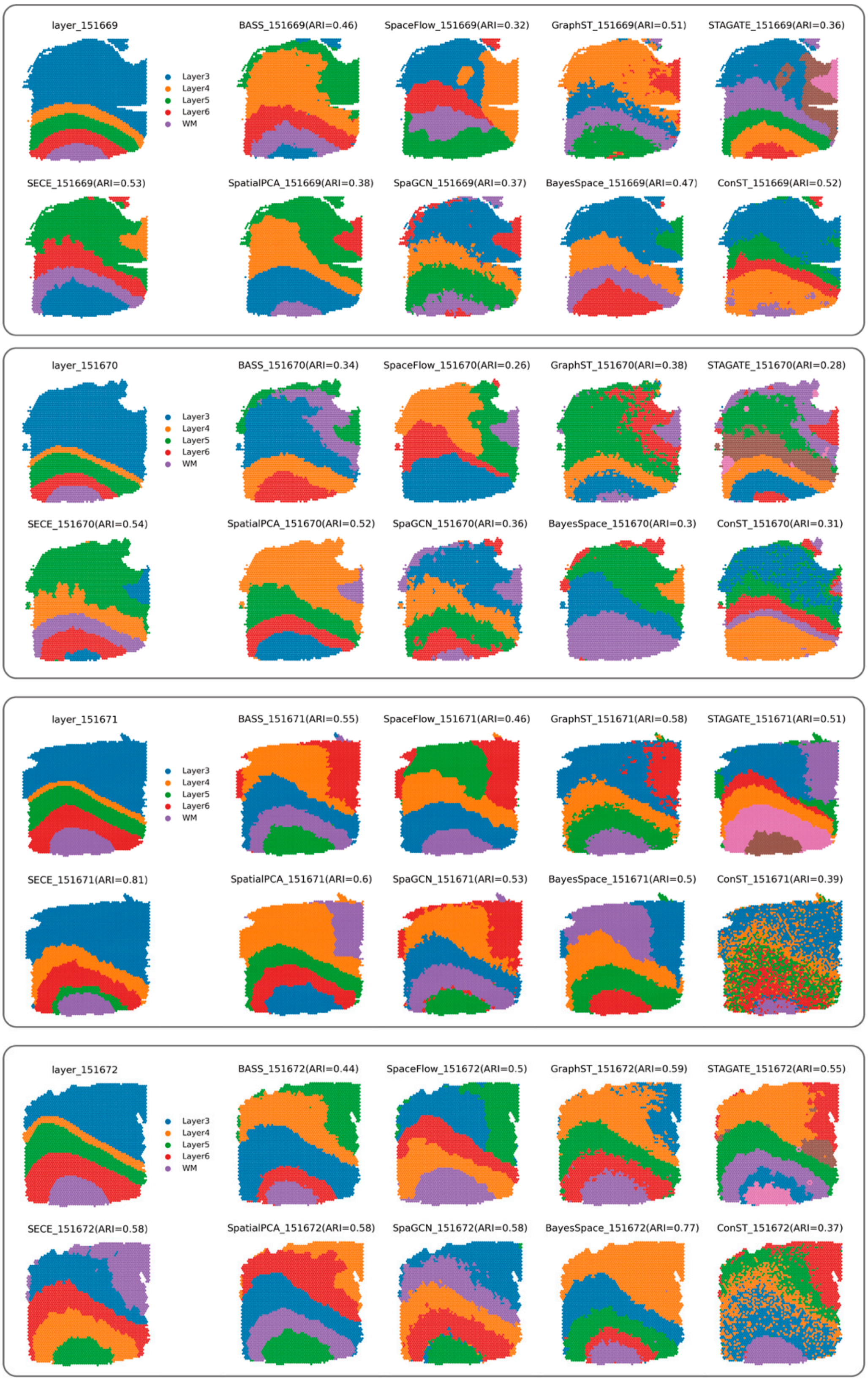

See next page

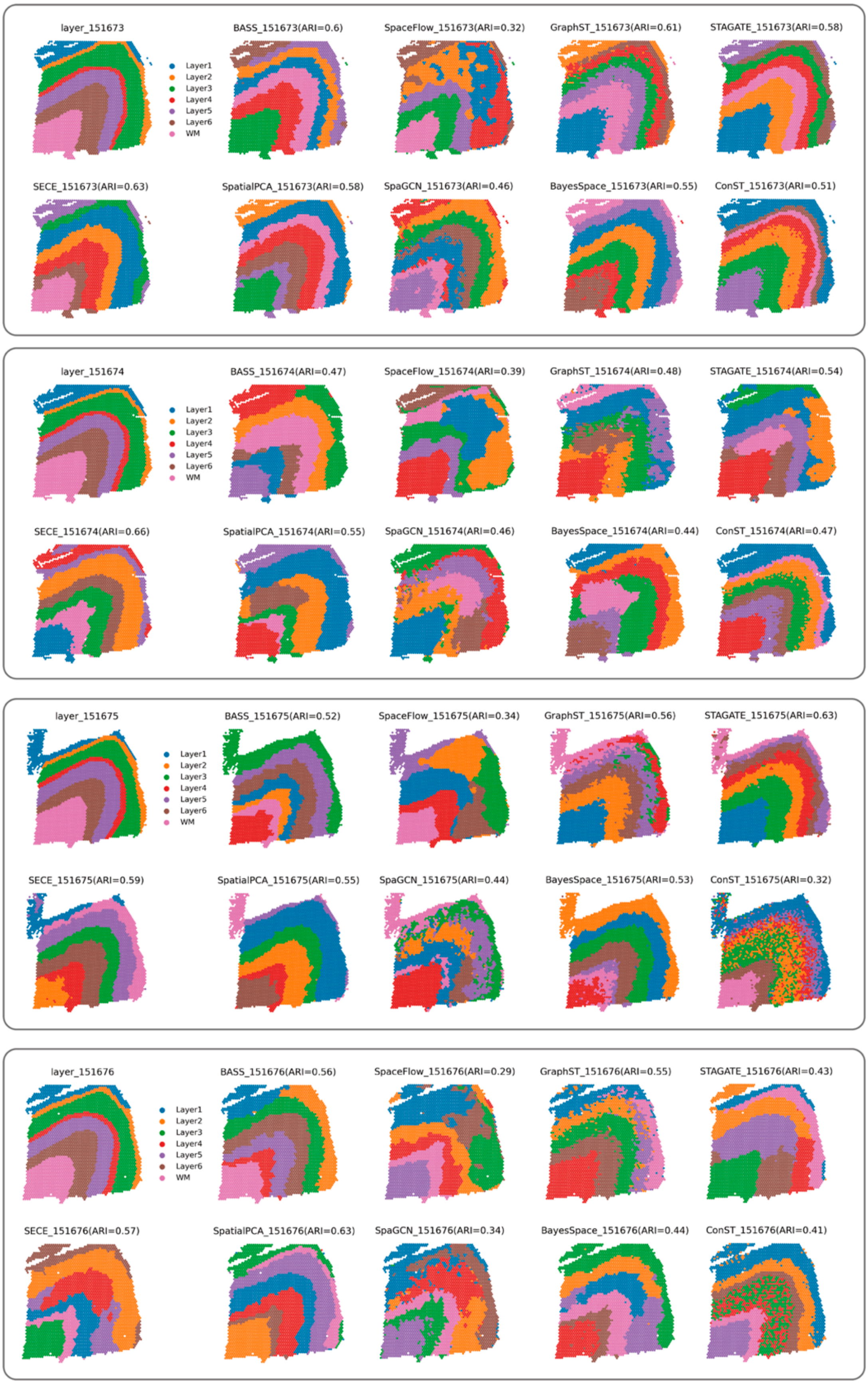

layer_151673
Layer1
Layer2
Layer3
Layer4
Layer5
Layer6
WM
BASS_151673(ARI=0.6)
SpaceFlow_151673(ARI=0.32)
GraphST_151673(ARI=0.61)
STAGATE_151673(ARI=0.58)
SECE_151673(ARI=0.63)
SpatialPCA_151673(ARI=0.58)
SpaGCN_151673(ARI=0.46)
BayesSpace_151673(ARI=0.55)
ConST_151673(ARI=0.51)
layer_151674
Layer1
Layer2
Layer3
Layer4
Layer5
Layer6
WM
BASS_151674(ARI=0.47)
SpaceFlow_151674(ARI=0.39)
GraphST_151674(ARI=0.48)
STAGATE_151674(ARI=0.54)
SECE_151674(ARI=0.66)
SpatialPCA_151674(ARI=0.55)
SpaGCN_151674(ARI=0.46)
BayesSpace_151674(ARI=0.44)
ConST_151674(ARI=0.47)
layer_151675
Layer1
Layer2
Layer3
Layer4
Layer5
Layer6
WM
BASS_151675(ARI=0.52)
SpaceFlow_151675(ARI=0.34)
GraphST_151675(ARI=0.56)
STAGATE_151675(ARI=0.63)
SECE_151675(ARI=0.59)
SpatialPCA_151675(ARI=0.55)
SpaGCN_151675(ARI=0.44)
BayesSpace_151675(ARI=0.53)
ConST_151675(ARI=0.32)
layer_151676
Layer1
Layer2
Layer3
Layer4
Layer5
Layer6
WM
BASS_151676(ARI=0.56)
SpaceFlow_151676(ARI=0.29)
GraphST_151676(ARI=0.55)
STAGATE_151676(ARI=0.43)
SECE_151676(ARI=0.57)
SpatialPCA_151676(ARI=0.63)
SpaGCN_151676(ARI=0.34)
BayesSpace_151676(ARI=0.44)
ConST_151676(ARI=0.41)

Figure S13. Spatial domains identified by SECE, BASS, SpaceFlow, GraphST, STAGATE, SpatialPCA, SpaGCN, BayesSpace, and conST, and manual annotation in 12 sections of the DLPFC dataset.

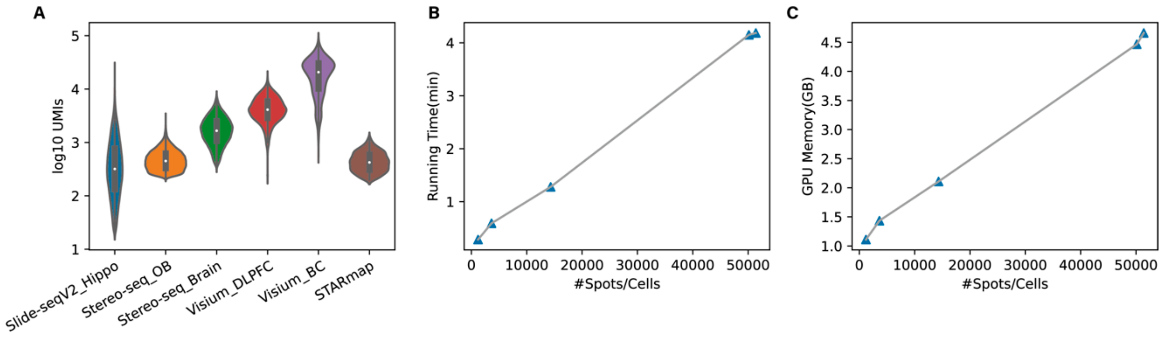

Figure S14. Datasets used by SECE and their running information. **(A)** Number of total UMIs per spot in each dataset. Running time **(B)** and GPU memory usage **(C)** on dataset with different numbers of spots based on NVIDIA® Tesla® V100 GPU.
